# Executive Function Improves Following Acute Exercise in Adults with Down Syndrome

**DOI:** 10.3390/brainsci11050620

**Published:** 2021-05-13

**Authors:** Shannon Ringenbach, Nathanial Arnold, Brandon Myer, Claire Hayes, Kahyun Nam, Chih-Chia Chen

**Affiliations:** 1 College of Health Solutions, Arizona State University, Tempe, AZ 85004, USA; nearnol1@asu.edu (N.A.); bmmyer@asu.edu (B.M.); chayes14@asu.edu (C.H.); nkhyun422@asu.edu (K.N.); 2 Department of Kinesiology, Mississippi State University, Starkville, MS 39762, USA; cc2196@msstate.edu

**Keywords:** intellectual disability, physical activity, weight training, cycling, cognitive function

## Abstract

The influence of exercise on brain function is an important topic, especially in persons with intellectual deficits. The aim of this study is to determine the effect of an acute bout of resistance training (RT) compared to assisted cycle therapy (ACT) and no training (NT) in adults with DS on cognitive function. Fourteen participants attended four sessions: a baseline assessment, an assisted cycling therapy (ACT) session, a resistance training (RT) session, and a session of no training (NT). In the RT session, the leg press, chest press, seated row, leg curl, shoulder press, and latissimus pulldown were performed. The ACT session consisted of 30 min of cycling and in NT session consisted of 20 min of board games. Inhibition was measured by the Erikson flanker task and cognitive planning was measured by the Tower of London test and both were administered prior to (pretest) and after each intervention (posttest). Our results showed that inhibition time improved more following RT and ACT than NT. There was also a significant difference between ACT and NT. For cognitive planning, improvements were seen following ACT and NT. In conclusion, an acute session of ACT demonstrated a significant trend towards improvements in executive functions in adults with DS which we interpreted using a model of neural changes and the cognitive stimulation hypothesis.

## 1. Introduction

Down syndrome (DS), also known as Trisomy 21, is the most common chromosomal condition in the United States. The Center for Disease Control (CDC) estimates that approximately 6000 babies, or 14 out of every 10,000 babies, are born with Down syndrome each year [1]. DS is the leading genetic cause of intellectual disabilities, and affects the behavioral and structural development for those with the disorder [1]. Additionally, DS causes moderate to severe cognitive impairment, including deficits in executive function (EF), as well as delays in language and speech development, nonverbal cognitive development, and auditory short-term memory [2,3]. Around 56.6% of adults with DS in the United States are employed, however executive functioning difficulties limit many everyday activities [4].

Executive functions are important to almost every aspect of life, and refer to a top-down mental process needed when you are concentrating and paying attention when automatic or instinct responses would be insufficient [5]. People with DS often have deficits in executive functioning, including inhibition, cognitive planning, attention, working memory, shifting, and processing speed [6]. Inhibitory control involves being able to control a person’s attention, behavior, and thoughts to override the internal or external prompt and then perform and alternative action [5,7]. Cognitive planning is commonly cited as a deficit in individuals with DS, yet it is rarely the topic of research or study [8,9]. Furthermore, cognitive planning is vital for independence (e.g., managing steps involved in transportation, jobs) and quality of life (e.g., planning involved in grooming, dressing etc.) in persons with Down syndrome.

Many people with DS often choose sedentary lifestyles and activities, which they themselves attribute to being too lazy, not having enough energy, or the skill being too difficult [10]. It has also been argued that inactivity among DS populations is shown through barriers such as lack of programs, transportation problems, family responsibilities, and lack of friends [11]. One solution is assisted exercise (AE), such as assisted cycle therapy (ACT), also known as forced exercise in animal studies, which is exercise that is helped by a mechanical motor that moves the limbs faster than they can on their own and has shown promising results for Parkinson’s disease (PD) patients [12,13,14] and has more recently been applied to the DS population [10].

In AE, the participant exercises at a higher rate than he or she would in voluntary exercise (VE), where the participant naturally selects his or her own cadence [13]. In the PD population, AE improved motor function, neuroprotective properties, and increased cerebral blood flow, which improved performance on cognitive tasks [12,13,14]. Assisted cycling therapy (ACT) is a form of AE, in which the participant pedals on a stationary bicycle with a motor. The motor enables the participant to pedal much faster than he or she would on his or her own [10]. Recently, acute sessions of assisted cycling therapy (ACT) compared to voluntary cycling or no cycling have been shown to improve cognitive planning, information processing, and improve manual motor functioning in adolescents with Down syndrome [10]. In a chronic ACT intervention with adolescents with DS, ACT improved inhibition and reaction times more than voluntary cycling and no cycling groups [15]. In an acute study with adults with autism spectrum disorder (ASD), inhibition also improved following ACT, but not voluntary or no cycling interventions [16]. There is a need for more research into the effects of an acute bout of ACT in connection with executive functions control within adults with DS.

Another form of exercise that has been researched in persons with DS is resistance training (RT). While there is some research concerning physical activity levels and performing tasks after RT in populations of DS, there is no research to our knowledge linking RT and cognitive function in adults with DS. There is evidence that RT does make young adults with DS more physically active [17], and has also been shown to increase daily functioning in persons with DS, with tasks such as walking up the stairs [18]. Resistance training for people with DS has also been shown to increase muscular endurance [19], muscular function [19], and physical activity engagement [17]. Research with typical adults has shown that resistance training increases EF. One research study showed how there was an increase in executive functioning in typically developed adults after a single session of resistance training when compared to their scores after the control session [20,21]. Since the above-stated studies reported that people with DS show similar physiological responses to typically developed individuals following RT, it would be expected that the cognitive function responses to resistance training would be similar to those seen in typically developed individuals.

To our knowledge, no studies have examined different exercise types in an acute setting in adults with DS and measured executive functions of inhibitory control and cognitive planning. The objective of this study was to determine if RT or ACT would equally improve executive functioning in adults with DS as measured by the Erickson flanker task and the Tower of London test. It was hypothesized that the inhibition time and cognitive planning would improve after the RT and ACT interventions but not the NT intervention. Furthermore, we hypothesized that RT due to its cognitive stimulation of having to plan about body position and which muscles to activate, would improve more than ACT which is more passive, and both exercise interventions would improve more than the no training intervention. This study will help gain a better understanding of the best intervention to increase executive functioning in the adults with DS. This study will also add to the limited information available with respect to RT effects on executive function in adults with DS.

## 2. Materials and Methods

### 2.1. Participants

Participants in this study were recruited through email announcements, fliers, and word of mouth among local organizations for people with Down syndrome. As can be seen in Table 1, there were a total of 14 adults each of whom completed all four sessions and had a mean mental age of 6.18 years. Previous research using a within-subjects acute design with adolescents with Down syndrome [10] and adults with autism [16] had sample sizes of 9 and 10 respectively and both found improvements in cognitive functioning following ACT compared to voluntary and no cycling which indicates that our sample size is appropriate. Refer to Table 1 for more participant characteristics. All protocols were approved by the Human Subjects Institutional Review Board of Arizona State University (HRP-503b). Each participant and/or their guardian (if appropriate) provided informed consent to participate in the research. 

### 2.2. Baseline

The first session included the baseline measure where consent and 2014 PAR-Q+ forms were completed to establish willingness and adequate health. Additionally, one repetition maximum (1RM) was assessed using weight-stack machines for the leg press, chest press, seated row, latissimus pulldown, shoulder press, and hamstring curl. 1-RM testing procedures followed the American College of Sports Medicine guidelines. The 1-RM for each of the exercises was then used to develop the resistant training plan for each participant, In addition, voluntary cycling speed, Peabody Picture Vocabulary Test (PPVT) IQ, ethnicity, height, weight, were determined at the baseline visit. The PPVT was used and is a commonly used method to determine the mental age of participants with DS [10,15].

### 2.3. Interventions

The participants were randomized through block randomization, into 3 different orders beginning with either resistance training (RT), assisted cycling therapy (ACT), or no training (NT) to avoid any validity threat due to the ordering of the interventions. The participants wore a Polar FT-7 TM heart rate device during each of the sessions to monitor their heart rate and rate of perceived exertion (RPE) was collected using a verbal scale (i.e., 1 = very easy, 2 = kind of easy, 3 = kind of hard, 4 = very hard). HR and RPE were recorded at the end of the warm-up period and after the conclusion of the final repetition of each exercise. The participants were tested pre- and post- intervention with a 10-min wait period between the intervention and post-testing which was used to reduce the influence of increased arousal and blood flow on cognitive function. Dependent measures were conducted randomly depending on the time of completion and availability of the other test. Once the first intervention was completed, the participants were randomized into a remaining protocol and the participant returned to the YMCA for the other two sessions. There were at least 48 h in between each of the sessions to limit a learning effect from repeated exposure. Participants were also asked to refrain from engaging in other physical activity outside of the study for the duration of their participation to eliminate confounds of other physical activity.

### 2.4. Resistance Training (RT)

This intervention began with a 5-min dynamic warm-up of high knees, hip circles, butt kickers, arm swings, and arm circles and then a 30-min resistance training session where the heart rate was recorded after each exercise was completed. Additionally, there was a one-minute wait period between each of the different exercises to reduce fatigue where the perceived exertion was collected from the participant. The exercises completed were leg press, leg curl, chest press, shoulder press, latissimus dorsi pull downs, and seated rows. Each exercise consisted of two sets with 8–12 repetitions at 75% of the participant’s 1RM until 16 repetitions of each exercise were completed. If a participant could not complete eight repetitions, the weight was lowered by 5% for the next set until a total of 16 repetitions was reached. The weight lifted and repetitions were recorded for each set of an exercise.

### 2.5. Assisted Cycle Therapy (ACT)

**Figure 1:**
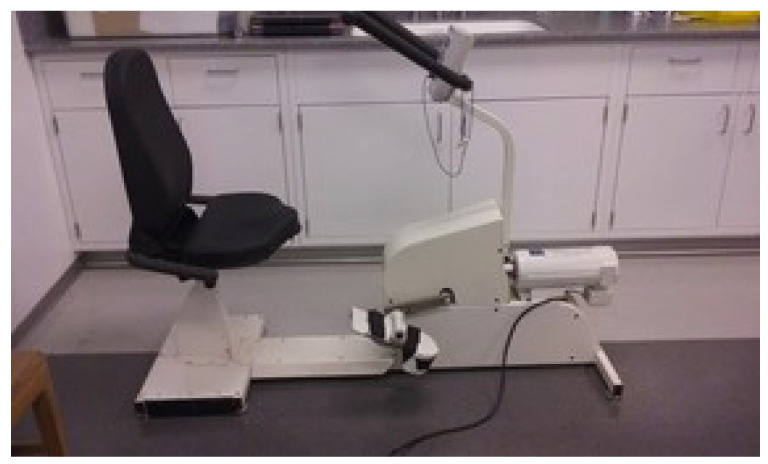
Experimental set up for assisted cycle Therapy (ACT).

Participants had a five-minute warm-up period where they would cycle at their own pace on the Theracycle recumbent bike (refer to Figure 1). Then the cadence of the bike was set to 135% of their voluntary speed from the baseline measurement and they continued cycling at the increased speed for 20 min. This protocol has been utilized frequently in other research with adolescents with DS [10,15] and adults with Parkinson’s disease [12]. The participant’s heart rate, cadence, and rate perceived exertion (RPE) were recorded every five minutes.

### 2.6. No Training (NT) or Control

The participants played a simple board game (i.e., Candyland or Chutes and Ladders) for 35 min. The heart rate and perceived exertion were recorded every five minutes during the intervention.

### 2.7. Dependent Measures

#### 2.7.1. Ericksen Flanker Task

This task was assessed using the website cognitivefun.net to test the participants’ inhibition control. The Flanker Task has been reported to be an applicable measure of executive function in older adults with mild cognitive impairment [22] and in kindergarten children who have the mental age of the present participants [23]. During the Erikson flanker task, participants are presented with a series of arrows. The arrow in the middle is either congruent or incongruent with the other arrows on the screen. Participants were to answer by tapping the left and right arrow button on the keyboard with the direction of the center arrow. The Erickson Flanker Task program recorded the percent correct, average congruent response time, and average incongruent response time. An inhibition time was calculated by finding the difference between the congruent and incongruent times. Lower inhibition times are indicative of better executive function performance.

#### 2.7.2. Tower of London Test (TOL)

The Tower of London (TOL) test used was designed to evaluate their cognitive planning abilities. This test was a subtest from the Developmental Neuropsychological Assessment [24] and has been supported by good validity evidence in persons with intellectual disability [25]. It utilized a wooden platform with three pegs of graduating height and three wooden balls (e.g., blue, red, and yellow). The goal of each trial was to move the balls from their starting positions to their final positions, as depicted by a printed image, in the given number of moves and within the 45-s time limit. Participants were instructed to move only one ball at a time. Each move ended with the ball on one of the three pegs which still had available space. After one practice trial, participants moved through 17 different trials of increasing complexity and difficulty. Each trial was considered a success if the participant achieved the goal arrangement in the provided number of moves and within the allowed 45 s. The test was ended if the participant failed to complete four trials in a row, either due to time limitations, failure to follow the rules, or completing the puzzle in too many moves. Thus, because the test is timed, the higher the total number of correct moves and the total number of attempts is related to better and faster cognitive performance.

### 2.8. Data Analysis

A repeated measure ANOVA of intervention (RT, ACT, NT) × Time (pre, post) was conducted on percent correct flanker, inhibition time flanker, total correct moves, and total number of attempts for TOL. Post hoc tests were conducted using separate one-tailed paired sample *t*-tests for significant dependent measures within SPSS version 26.

In addition, the participants were also randomized through block randomization into different orders of the interventions (e.g., A = RT, ACT, NT; B = ACT, NT, RT, C = NT, RT, ACT) and an ANOVA on delta scores did not reveal any significant differences due to order of intervention.

## 3. Results

Based on a one way ANOVA of mean RPE for all RT tasks, and for ACT and NT, there were no significant differences *p* > 0.26 among these interventions, however a one-way ANOVA for HR was significant F(2,38) = 12.18, *p*= 0.00 and post hoc analysis showed that RT HR was greater than NT (*p* = 0.00) and was tending towards greater than ACT (*p* = 0.082). HR for ACT was also greater than HR for NT (*p* = 0.033). 

### 3.1. Inhibition

**Figure 2:**
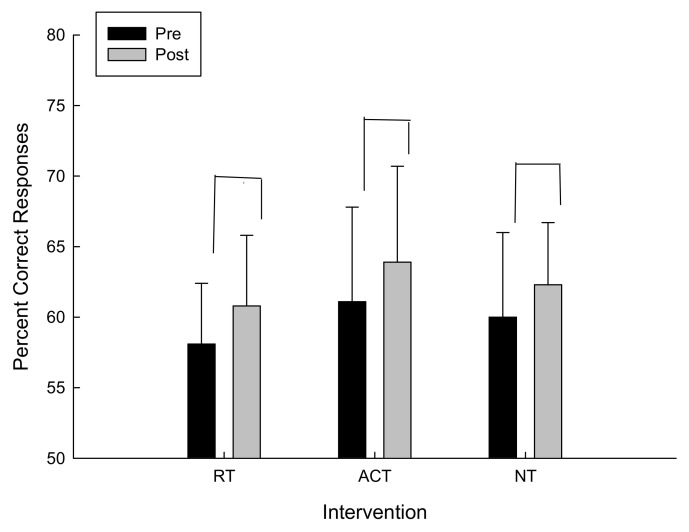
Percent correct responses as a function of intervention and time.

For the percent correct responses there was a trend towards conventional levels of significance for the main effect of time F(1,12) = 1.062, *p* = 0.16 in which the percent correct responses increased in all interventions (refer to Figure 2).

**Figure 3:**
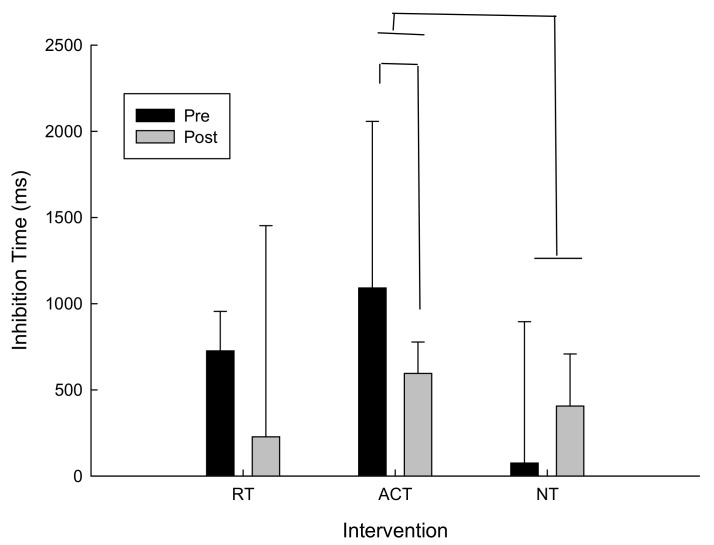
Inhibition time (incongruent–congruent) as a function of intervention and time.

For delta scores (pre-post) for inhibition time (i.e., incongruent response time–congruent response time) there was a trend towards conventional levels of significance for the main effect of time following ACT, t(13) = −1.1, *p* = 0.15. Post hoc analysis revealed that inhibition time decreased which suggests improved performance following ACT. As can be seen in Figure 3, inhibition time also improved more following ACT than NT. In fact, following NT inhibition times increased, whereas inhibition times decreased following ACT and RT. It is important to note that variability was high in this task. However because this is a within-subjects design and the participants were randomized into three different intervention orders that did not affect the results based on the analysis described in the data analysis section, we are confident that any differences that may appear in the baseline measures were accounted for.

### 3.2. Cognitive Planning

**Figure 4:**
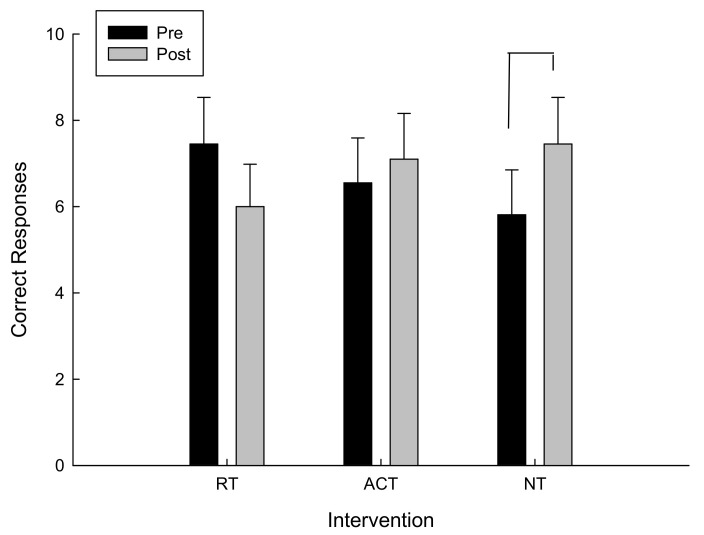
Correct moves as a function of time and intervention.

For the total number of correct moves, there was a significant interaction between intervention and time F(2,20) = 3.08, *p* = 0.034. As can be seen in Figure 4 and confirmed by post hoc analysis, for the total number of correct moves, there was a main effect of time (pre/post) following the NT intervention, t(10) = −1.99, *p* = 0.038, however there were no significant differences following RT and ACT. The higher the total correct moves, the farther the participant went in the testing protocol and the better the performance.

**Figure 5:**
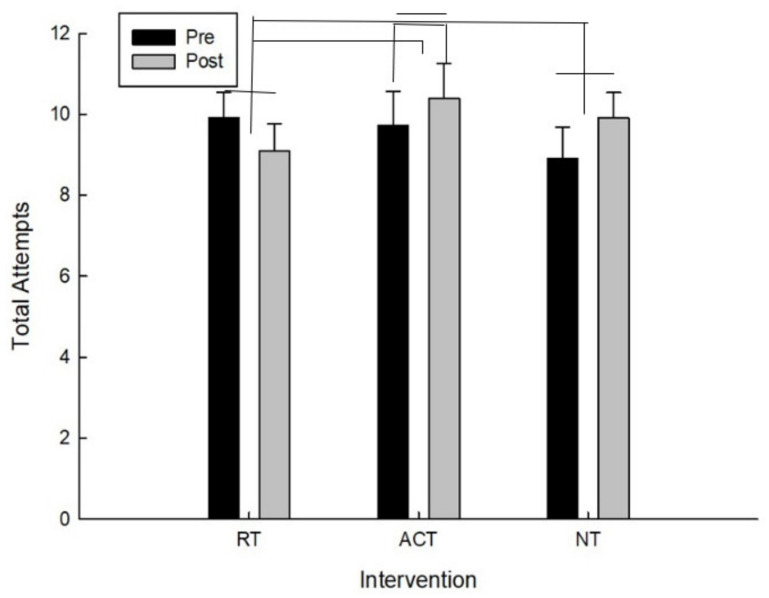
Total attempts as a function of time and intervention.

For the total number of attempts, there was a significant interaction between intervention and time F(2,20) = 5.09, *p* = 0.020. As can be seen in Figure 5 and confirmed by post hoc analysis, for the total number of attempts, the main effect of time (pre/post), following the ACT intervention, t(10) = −1.55, *p* = 0.076 approached conventional levels of significance in which the total attempts increased. The higher the total number of attempts means that they went farther on the testing protocol and because this is a timed test it also means they were making decisions faster.

For the total number of attempts there was also a significant difference for improvement (pre-post) between RT and ACT, t(10) = −2.14, *p* = 0.029 and between RT and NT, t(10) = −2.09, *p* = 0.032 which is because total attempts increased for both ACT and NT whereas they decreased following RT.

## 4. Discussion

This is the first study, to our knowledge, that has examined an acute exercise interventions comparing resistance training (RT), assisted cycle therapy (ACT) and no training/playing games (NT), and measured inhibition and cognitive planning in adults with DS. Improving inhibition and cognitive planning are essential to activities of daily living, maintaining independence and employment. For example, inhibition is important for health in that we must inhibit bad food choices and this is especially important in adults with DS who have a high rate of obesity. Inhibiting inappropriate reactions to situations is also important for adults with DS when they are performing their jobs or are in social situations. Improving cognitive planning is also essential to activities of daily living, for example, cognitive planning is important for grocery bagging in which you would plan which groceries to put on the bottom and how to stack them. Cognitive planning is also important for planning out the different steps needed to use public transit system, for dressing/grooming appropriately, etc.

### 4.1. Neural Hypothesis

Our results are consistent with our hypothesis that the inhibition time would decrease more with ACT than NT. Our results showed that while inhibition time improved following ACT and RT and it was only significantly different between ACT and NT. This is consistent with previous research in another acute intervention study in which adults with autism spectrum disorder (ASD) improved inhibition following an acute ACT intervention but not voluntary cycling or no cycling interventions [15]. In a chronic 3×/week for 8 weeks ACT intervention with adolescents with DS, ACT also improved inhibition and reaction times more than voluntary cycling and no cycling groups [15].

For cognitive planning, our results are also consistent with our hypothesis that ACT would have a greater effect than no exercise (NT) on cognitive planning, however cognitive planning also improved following NT and became worse following RT which were inconsistent with our hypotheses. The improvement following ACT is consistent with our research in adolescents with DS in a chronic intervention of 3×/week for 8 weeks in which ACT improved cognitive planning using the same measure as this study, more than voluntary cycling or no cycling [26]. Hozapfel and colleagues (2015) [26] also found improvements in the assembly task of the Purdue pegboard test, which measures manual dexterity following ACT but not VC or NC. This may be because the assembly task requires placing a peg in a hole with the right hand, then a washer with the left hand, then a collar with the right hand, then a washer with the left hand. Clearly this requires some cognitive planning to get the order correct. Other chronic exercise intervention studies with adolescents with DS found improvements in other measures of executive function of set-shifting, verbal fluency, reaction time, and inhibition following eight weeks of ACT [15,23]. The improvements following NT in this study are not consistent with our past research, however in the past we have used a no-exercise group that did not come into the lab [15] or watched a video in our acute studies [10]. The no-training session in the present study played board games, which may have involved more cognitive planning and arousal than our previous control conditions.

**Figure 6:**
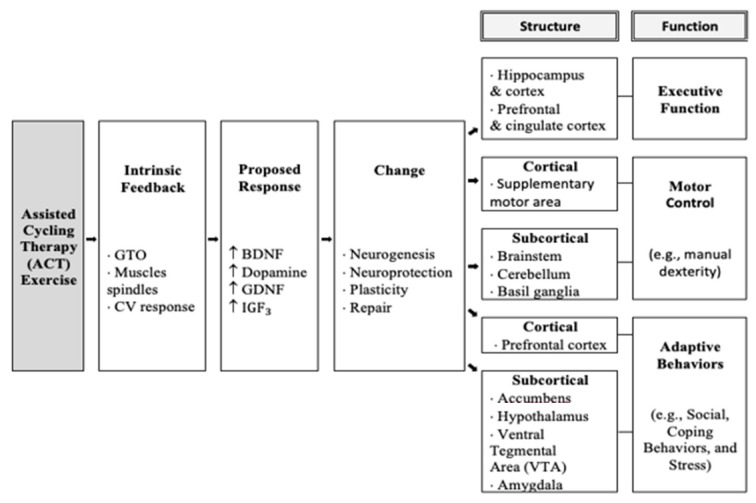
Model of mechanisms of Assisted Cycle Therapy.

Taken together, the most significant improvements were seen following the ACT intervention. This was consistent with previous research with adolescents with DS following acute [10] and chronic [15] ACT interventions. The explanation for improved executive function following ACT can be seen in Figure 6 in the model below and shows that the high rate of movement triggers afferent information to be sent to the prefrontal cortex, which triggers the release of brain-derived neurotrophic factors (e.g., BDNF, IGF, GDNF, domapine, which have been shown to improve executive function. This is consistent with previous research in animal studies of Parkinson’s disease, as it was suggested that the high rate of cycling may “trick” the body into believing it is exercising at a high intensity necessary to release neurotrophic factors and create changes in the brain [12]. The model below, modified by Ringenbach and colleagues, shows ACT initiates an intrinsic feedback loop leading to increased production of neurotrophic factors thought to cause structural and functional changes and ultimately long term executive function improvements.

### 4.2. Cognitive Stimulation Hypothesis

The cognitive stimulation hypothesis states that physical exercise should be cognitively demanding in order to challenge the higher-order cognitive processes in order to induce significant improvements to cognitive functioning [27,28]. We predicted that because of the increased cognitive requirements of resistance training, executive function may improve more with RT than ACT and NT. We did find that RT slightly improved inhibition. Previous research had shown that acute bouts of RT had a large effect size for the cognitive test of inhibition in healthy adults (ES = 0.73) compared to no-exercise groups [29]. Research from Landrigan and colleagues [30] determined that individuals with unspecified cognitive impairment saw the most improvement in tests of executive function following a minimum of four weeks of RT in typical adults. Research in the typical population has shown that acute sessions of RT increased executive functioning significantly [31]. However, in our study we used weight machines instead of free weights for the safety of our participants, which may have eliminated the need for cognitive planning during RT. According to Best in 2010, more cognitively engaging exercises tend to have a more preferable effect on executive function, in typically developing children, in contrast to less-engaging exercises and our results show this is true for adults with DS. Recent research has shown that exercises that target balance have improved cognitive performance in typical middle-aged adults [32,33]. Future research should investigate exercise involving balance on cognitive function in adults with Down syndrome.

Furthermore, we were surprised to see trends for improvements following NT. In previous research [10,15], the ACT exercise group was compared to a no exercise group that continued their current lifestyle and did not come to the lab and did not show any improvements on any measures pre/post. However, in this study we used a NT group in which the games chosen to play did involve some counting and moving game pieces, which involved cognitive planning. Due to our participants having a mean mental age of 6.2 years, we believe that the games involved enough executive functioning, specifically cognitive planning (e.g., counting, moving) that some of the measures improved after the NT intervention because of an increased cognitive workload. One previous study found that typical children, aged 10–12 years old, had a significant increase in some executive functions (e.g., updating and shifting) after a 6-week trial of card and board games [34]. Thus cognitive stimulation could be another explanation of our results, especially with respect to the improvements following NT.

### 4.3. Intensity Hypothesis

Another explanation for the improvement of ACT was that the intensity of cycling may have been at the right rate for improvements in executive functioning. With regard to intensity, the inverted-U hypothesis has been noted in prior studies [25]. This hypothesis states that lower and higher exercise intensities will not have as much of an effect on executive functioning in comparison to moderate intensity exercises. In an acute study on cognitive functioning and exercise intensity in adults with DS, moderate-intensity exercises were beneficial for improving inhibitory control [35]. As can be seen in Table 2, ACT had moderate heart rates with RT having higher and NT having lower heart rates. This may be another explanation as to why the ACT intervention increased the executive functioning tasks more than the other interventions in this study.

## 5. Conclusions

Our results could be generalized to suggest that activity that involves cognitive functioning or triggers neurotransmitters will improve executive function, specifically inhibition and cognitive planning. For example, parents, therapists, and teachers should pay close attention to the cognitive aspect of activities to enhance learning, which will ultimately improve their job opportunities and quality of life.

## Figures and Tables

**Table 1:** Participant Characteristics.

	Mean		Standard Deviation	
Chronological Age	26 years 3 months		5 years 2 months	
Mental Age	6 years 2 months		4 years 5 months	
BMI	33.6		9.12	
Sex	Male		Female	
	8		6	
Ethnicity	White	African American	Hispanic	Native American
	10	1	2	1

**Table 2:** Task Parameters.

Resistance Training								
	Total Repetitions		Percent of Max (%)		Heart Rate (BPM)		RPE	
	M	SD	M	SD	M	SD	M	SD
Leg Press	16.57	1.59	70.18	5.17	100.21	15.14	1.79	0.77
Chest Press	16.79	1.78	68.92	6.23	104.64	17.14	2.14	1.06
Lat Pulldown	17.14	2.47	73.04	10.78	102.93	14.63	2.43	1.12
Seated Row	17.14	1.96	67.76	7.72	104.71	17.65	2.29	0.88
Leg Curl	16.71	1.79	71.12	10.42	104.93	19.19	2.43	1.12
Shoulder Press	16.79	1.61	67.67	10.31	108.36	19.10	2.64	1.17
Assisted Cycling Therapy								
	Average Cadence (RPM)		% of Voluntary Cadence		Heart Rate (BPM)		RPE	
ACT	64.36	22.53	128	16	91.31	17.08	1.5	0.66
No Training								
					Heart Rate (BPM)		RPE	
NT					75.88 *	9.82	1.64	0.71

* *p* < 0.05 for NT Heart rate compared to all RT.

## Data Availability

Data available on request due to ethical restrictions.
